# Adverse associations between maternal deoxynivalenol exposure and birth outcomes: a prospective cohort study in China

**DOI:** 10.1186/s12916-023-03011-5

**Published:** 2023-08-28

**Authors:** Tianqi Tan, Tingting Chen, Wenwen Zhu, Lin Gong, Yizhong Yan, Qian Li, Li Chen, Yiling Li, Jialin Liu, Yanan Li, Xuefeng Yang, Liping Hao, Huaiji Wang, Nianhong Yang, Sheng Wei

**Affiliations:** 1 Department of Nutrition and Food Hygiene, Hubei Key Laboratory of Food Nutrition and Safety, School of Public Health, Tongji Medical College, Huazhong University of Science & Technology, 13 Hangkong Road, Wuhan, 430030 Hubei China; 2 Department of Epidemiology and Biostatistics, School of Public Health, Tongji Medical College, Huazhong University of Science & Technology, Wuhan, 430030 Hubei China; 3 Wuhan Center for Disease Control and Prevention, Institute of Environmental Health, 288 Machang Road, Wuhan, 430022 Hubei China; 4 School of Public Health and Emergency Management, Southern University of Science and Technology, Shenzhen, 518055 Guangdong China

**Keywords:** Deoxynivalenol, Birth weight, Small for gestational age, Pregnant women, Cohort study

## Abstract

**Background:**

Deoxynivalenol (DON), one of the most prevalent mycotoxins, has been found to cause fetal growth retardation in animals. However, limited evidence exists regarding its effects on pregnant women.

**Methods:**

Maternal urinary concentration of total DON (tDON) and free DON (fDON) in the second trimester was measured using liquid chromatography with tandem mass spectrometry. Provisional daily intake (PDI) of DON was calculated based on tDON concentration. Linear and logistic regression models were used to evaluate the association between DON exposure levels and birth weight, birth length, and the risk of small for gestational age (SGA).

**Results:**

Among 1538 subjects, the median concentrations of tDON and fDON were 12.1 ng/mL and 5.1 ng/mL, respectively. The PDI values revealed that the median DON intake was 0.7 µg/kg bw, and 35.9% of the total population exceeded the provisional maximum tolerable daily intake (PMTDI) of 1 µg/kg bw. Compared with the lowest tertile, birth weight decreased by 81.11 g (95% CI: -127.00, -35.23) for tDON (*P*-trend < 0.001) and 63.02 g (95% CI: -108.72, -17.32) for fDON (*P*-trend = 0.004) in the highest tertile. Each unit increase in Ln-tDON and Ln-fDON was also inversely associated with birth weight. Furthermore, compared to those who did not exceed PMTDI, pregnant women whose PDI exceeded PMTDI had lower birth weight (β = -79.79 g; 95% CI: -119.09, -40.49) and birth length (β = -0.21 cm; 95% CI: -0.34, -0.07), and a higher risk of SGA (OR = 1.48; 95% CI: 1.02, 2.15) in their offspring. Similar associations with birth weight, birth length, and SGA were found when comparing the highest tertile of PDI to the lowest tertile (all *P*-trend < 0.05).

**Conclusions:**

Maternal DON exposure is related to decreased birth weight. Our findings implicate that DON exposure during pregnancy may cause fetal growth faltering, and measures should be taken to reduce DON exposure in pregnant women.

**Supplementary Information:**

The online version contains supplementary material available at 10.1186/s12916-023-03011-5.

## Background

Deoxynivalenol (DON) is a type of mycotoxin produced by *Fusarium* species commonly found in cereal crops such as wheat, barley, and maize [1, 2]. DON is known to be stable even after food processing, leading to high levels of human exposure [3, 4]. Acute toxicities of DON mainly include nausea, vomiting, diarrhea, and feed refusal. Chronic exposure can lead to growth faltering, neurological, as well as immunological dysfunction in animals, while the effects of long-term exposure on humans have not been reported [5, 6]. Considering the high exposure level and multisystem toxicity of DON, both the Joint Expert Committee on Food and Additives (JECFA) and the European Food Safety Authority (EFSA) established a provisional maximum tolerable daily intake (PMTDI) for DON at 1 µg/kg bw/day [7, 8].

Dietary DON exposure varies across populations and regions. As one of the largest agricultural producers, China is particularly vulnerable to DON contamination in food [9]. In 2017, Yan et al. analyzed DON in wheat and corn samples collected in China. The results showed that all wheat samples were positive for DON, and 99.83% of corn samples were positive. The mean concentrations of DON were found to be 165.87 μg/kg in wheat and 175.30 μg/kg in corn [10]. Recent studies have also reported high levels of DON exposure in subjects from Henan province in China, with more than half of individuals exceeding the PMTDI [11, 12]. According to Chinese dietary guidelines from 2016, pregnant women are advised to increase their intake of foods and nutrients during the second and third trimesters to meet the nutritional needs of both the mother and the fetus. Therefore, the high levels of DON in cereals may contribute to increased dietary exposure in pregnant women, potentially leading to adverse health effects.

Although DON has been reported to increase the health risk of animals during pregnancy, its effects on human health are not well understood. Studies have shown that DON can cross the placental barrier in both animals and humans, maternal dietary exposure leads to fetal DON exposure [13–15]. In pregnant sows, maternal DON exposure has been linked to growth retardation in offspring, with lower doses affecting growth more than appetite [16]. Several studies using grain farming and weather conditions as surrogate indicators have found that occupational exposure to mycotoxins, including DON, was associated with an increased risk of adverse pregnancy outcomes [17]. While other mycotoxins, such as aflatoxin, have been suggested to impair intrauterine fetal growth and lead to lower birth weight [18], there are few studies on the effects of DON exposure during pregnancy. Collectively, it is worthwhile to explore the potential adverse effects of DON exposure in pregnant women on birth outcomes.

Thus, to obtain more extensive data on DON exposure in pregnant women and further understand its toxicity, we assessed the association between maternal DON exposure during pregnancy and birth outcomes in the Tongji Maternal and Child Health Cohort (TMCHC) in Wuhan, China.

## Methods

### Study design

All subjects were drawn from the TMCHC, a population-based prospective cohort study primarily designed to explore maternal nutrition, lifestyle, and environmental effects on the health and disease of mother-infant pairs in Wuhan, Hubei Province, central China [19]. In TMCHC, pregnant women were enrolled prior to 16 weeks of gestation and followed up across prenatal periods. We restricted the analyses to participants who were live singleton births and donated urine samples before 28 weeks of gestation. Considering over 83.0% of pregnant women have morning sickness during early pregnancy, we excluded women with urine samples collected at less than 16 weeks. Finally, 1538 pregnant women were involved in analyses in the present study (Additional file 1: Fig. S1).

The study was approved by the ethics review committee of Tongji Medical College of Huazhong University of Science and Technology in China (NO. 201302). Written informed consent was obtained from each participant at enrollment.

### Urinary DON test

Urinary free DON (fDON) and total DON (tDON) were determined using the ultra-performance liquid chromatography-tandem mass spectrometry (UPLC-MS/MS) method, and the detailed descriptions have been published previously [20]. Dietary DON intake is usually excreted in the urine as fDON and DON-glucuronides in humans, tDON is sum of free DON and DON-glucuronides. DON may not be detected in plasma due to the rapid clearance of DON from blood [21]. Therefore, we tested the urinary DON levels for assessing human exposure to DON.

In brief, an internal standard (^13^C_15_-DON) was added to a mixture of 500 μL urine sample and 1.5 mL water, resulting in a final concentration of 10 ng/mL. For the measurement of tDON, the urine samples were first treated with 200 μL phosphate buffer containing 1000 Units of β-glucuronidase and incubated in a shaker incubator at 37 °C for 16 h. After centrifugation, urine samples were cleaned and enriched using a solid-phase extraction column. Detection was performed on UPLC-MS/MS Xevo TQS system (Waters, MA, USA) equipped with an ACQUITY UPLC HSS T3 column (2.1 mm × 100 mm, 1.8 μm, Waters, MA, USA). The mobile phase A consisted of 5 mmol/L ammonium acetate, and B was methanol. The flow rate was set at 0.2 mL/min with a volume injection of 10 µL, and the total run time was 8 min. One blank and one standard solution with 10 ng/mL of DON were added in each batch of 30 samples as the negative and positive control, respectively. The limit of detection (LOD) was 0.5 ng/mL of urine, and values below the LOD were set to half of the LOD for analysis. The laboratory personnel were blind to the subject information of the urine sample.

### DON exposure assessment

The provisional daily intake (PDI) of DON was estimated based on the urinary DON biomarker levels using the following equation:

PDI (μg/kg bw) = C × V × 100/ (W × E).

where C = total DON concentration (μg/L), V = daily urine volume (L), W = body weight of participant (kg) and E = urinary excretion rate of DON (%). In the calculation, daily urine excretion was assumed to be 2 L for pregnant women [22]. A daily urinary DON excretion rate of 68% was used during the calculation of PDI [23].

### Birth outcomes

Information on the mode of delivery (vaginal or cesarean delivery), date of birth, and infant sex was obtained from birth records at the hospital. Gestational age at delivery was calculated based on the last menstrual period and the date of birth. For women who could not accurately recall their last menstrual period or had irregular menstrual cycles, ultrasound examination was used to confirm the last menstrual period. Birth weight and length were measured by a trained nurse in the delivery room.

Small for gestational age (SGA) was defined as weight < 10th percentile adjusted for gestational age and sex, based on all newborns in the TMCHC from Wuhan, China. Low birth weight (LBW) was defined as birth weight < 2.5 kg, regardless of gestational age or sex. Preterm birth (PTB) was defined as being born alive before 37 weeks of gestation.

### Covariates

Information on maternal socio-demographic characteristics and lifestyle factors was obtained from a standardized and structured questionnaire at enrollment, including maternal age, average personal income (< 5000, 5000–9999, ≥ 10,000 RMB yuan/month), education years (≤ 12, 13–15, ≥ 16 years), parity, abnormal pregnancy-labor history (yes/no), morning sickness (yes/no), alcohol intake (yes/no), smoking (yes/no). Maternal weight and height were measured at the time of enrollment and repeated in follow-up visits. Pre-pregnancy body mass index (BMI, kg/m^2^) was calculated using self-reported pre-pregnancy weight and height measurement. Dietary intake during the second trimester was assessed using a validated food frequency questionnaire [24]. Based on dietary records, food groups that provide the main source of cereals in the Chinese diet were identified, including rice products, flour and its products, coarse cereals. The season of urine sampling was divided into spring (March to May), summer (June to August), autumn (September to November), and winter (December to February), according to the sampling date. Urinary creatinine was detected using a sarcosine oxidase method (Mindray BS-200 creatinine kit, Shenzhen Mindray Bio-medical Electronics Co., Ltd) [25].

### Statistical analysis

Descriptive statistics were presented as means ± standard deviations (SD), medians, and 95% ranges. For statistical tests, undetectable DON biomarker concentration was set as half the LOD. Spearman’s rank correlation test was used to assess the correlations between total DON and free DON.

Linear regression models were used to estimate the association between maternal DON exposure and birth weight and birth length. Binary logistic regression models were used to identify whether maternal DON exposure was associated with the risk of SGA, LBW, and PTB. Models were fit using DON exposure as a continuous variable through the natural logarithm transformed (Ln-tDON, Ln-fDON, Ln-PDI) or as categorical variables (tertiles). Tertiles were defined based on the distribution of DON exposure levels, with the lowest tertile (T1) as the reference. To quantify a linear trend, the median values for each tertile were treated as a continuous variable in the regression model. Besides, the provisional daily DON intake was categorized into a dichotomous variable using the PMTDI of 1 μg/kg bw/day as the cut-off point. We also used the creatinine-corrected DON levels to explore the association between DON and birth outcome to account for variability in urine dilution between individual samples. In addition, stratified analyses were performed by maternal age (< 28, ≥ 28 years) and infant sex (boys, girls), with tested interaction by modeling cross-product terms in the regression model.

All multivariable-adjusted models included maternal age, pre-pregnancy BMI, weight, average personal income, education years, parity, abnormal pregnancy-labor history, morning sickness, alcohol intake, smoking, the season of sample collection, infant sex, and gestational age at delivery. Moreover, we performed the sensitivity analysis further adjustments for cereal intake among women for whom dietary data were available (*n* = 712). Missing values of classification variables were encoded as the missing indicator category for analysis. *P* values < 0.05 were considered to be statistically significant. Statistical analyses were conducted using SAS, Version 9.4 (SAS Institute).

## Results

### Demographics and urinary DON levels

Table 1 presents the maternal, fetal characteristics, and urine DON levels. A total of 1538 mother-infant pairs were included in the current analysis, the mean age of mothers was 28.3 ± 3.4 years and the average pregnancy-BMI was 20.9 ± 2.7 kg/m^2^. Among the infants, the average birth weight was 3334.9 ± 445.4 g, birth length was 50.1 ± 1.5 cm, and the incidence of SGA was 8.5%, LBW was 2.4%, PTB was 4.0%. Major characteristics of the study population were similar to subjects whose urine was available and unavailable before 28 weeks. (Additional file 1: Table S1).

**Table 1:** Subject characteristics (*n* = 1538)

	Mean ± SD, median (95% range), or *n* (%)		Mean ± SD, median (95% range), or *n* (%)
**Maternal characteristics**		**Urinary DON examination**	
Maternal age (years)	28.3 ± 3.4	Season of sample collection	
Pre-pregnancy BMI (kg/m2)	20.9 ± 2.7	Spring	301 (19.6)
Weight (kg)	54.8 ± 7.9	Summer	476 (31.0)
Average personal income (RMB yuan/month)		Autumn	525 (34.1)
< 5000	533 (34.7)	Winter	236 (15.3)
5000–9999	675 (43.9)	Creatinine (g/L)	1.0 ± 0.6
≥ 10,000	304 (19.8)	Total DON	
Missing	26 (1.7)	Positive Samples	1487 (96.7)
Education attainment (years)		Mean value (ng/mL)	19.0 ± 20.4
≤ 12	213 (13.9)	Median value (ng/mL)	12.1 (1.1, 59.5)
13–15	388 (25.2)	Mean value (ng/mg Creatinine)	20.1 ± 25.2
≥ 16	886 (57.6)	Median value (ng/mg Creatinine)	13.5 (1.8, 56.4)
Missing	51 (3.3)	Free DON	
Parity (Primiparous)	1271 (82.6)	Positive Samples	1324 (86.1)
Abnormal Pregnancy-Labor History (Yes)	570 (37.1)	Mean value (ng/mL)	10.2 ± 13.2
Morning sickness (Yes)	1276 (83.0)	Median value (ng/mL)	5.1 (ND, 36.2)
Smoking (Yes)	99 (6.4)	Mean value (ng/mg Creatinine)	9.9 ± 12.8
Alcohol intake (Yes)	71 (4.6)	Median value (ng/mg Creatinine)	5.9 (0.4, 29.9)
**Infant characteristics**		PDI (ng/kg b.w.)	
Sex (male)	886(57.6)	Mean value	1.0 ± 1.1
Gestational age (weeks)	39.4 ± 1.4	Median value	0.7 (0.1, 3.2)
Birth weight (g)	3334.9 ± 445.4	Exceeding PMTDI	552 (35.9)
Birth Length (cm)	50.1 ± 1.5		
SGA (< 10%)	130 (8.5)		
Low birth weight (< 2,500 g)	37 (2.4)		
Preterm birth (< 37 weeks)	61 (4.0)		

*Abbreviations*: *SD* standard deviation, *BMI* body mass index, *SGA* small for gestational age, *DON* deoxynivalenol, *ND* none detected, *PDI* provisional daily intake, *bw* body weight, *PMTDI* provisional maximum tolerable daily intake

Total DON was detected in 96.7% of the urine samples, and the median concentration was 12.1 ng/mL or 13.5 ng/mg creatinine. Free DON was detectable in 86.1% of the urine samples, and the median concentration was 5.1 ng/mL or 5.9 ng/mg creatinine. Urinary tDON levels were significantly correlated with the levels of fDON (Additional file 1: Fig. S2, *r* = 0.810, *P* < 0.001). Using the urinary biomarker analysis data, the median PDI of DON among all participants was 0.7 µg/kg bw, and 552 of the 1538 participants (35.9%) exceeded the PMTDI of 1 µg/kg bw.

### The associations between urinary DON levels and birth outcomes

The associations between urinary tDON, fDON levels and birth outcomes are presented in Table 2. After adjustment for potential confounders, per-unit increment of ln-transformed urinary tDON levels was related to decreased birth weight (adjusted β = -22.00 g, 95% CI: -37.11, -6.89). Compared to the lowest tertile of tDON concentration, birth weight decreased by 81.11 g (95% CI: -127.00, -35.23) in the highest tertile (*P* for trend < 0.001). Similarly, a significant dose-related association of fDON concentration with lower birth weight [adjusted β for Ln-fDON = -17.70 g (95% CI: -29.72, -5.69); T3 vs. T1 = -63.02 g (95% CI: -108.72, -17.32); *P* for trend = 0.004] was found. Moreover, we performed the analysis using creatinine-adjusted DON levels, and the results showed that the association between urinary DON and birth weight remained statistically significant (Additional file 1: Table S2). Urinary tDON and fDON levels were not associated with the birth length and the risk of SGA (Table 2), LW, and PTB (Additional file 1: Table S3).

**Table 2:** Associations of maternal urinary DON levels (ng/mL) during pregnancy with birth outcomes

	**Birth weight (g)**	**Birth length (cm)**	**Small for gestational age**	
	β (95%CI)	β (95%CI)	Case (%)	OR (95%CI)
**Total DON**				
Ln-tDON	-22.00 (-37.11, -6.89)	-0.03 (-0.08, 0.03)	130 (8.45)	1.09 (0.94, 1.27)
T1	ref	ref	37 (7.23)	ref
T2	-21.42 (-67.01, 24.13)	0.08 (-0.08, 0.24)	44 (8.53)	1.28 (0.84, 2.04)
T3	-81.11 (-127.00, -35.23)	-0.15 (-0.31, 0.01)	49 (9.61)	1.44 (0.91, 2.28)
* P* for trend	< 0.001	0.021		0.149
**Free DON**				
Ln-fDON	-17.70 (-29.72, -5.69)	-0.04 (-0.08, 0.01)	130 (8.45)	1.10 (0.97, 1.24)
T1	ref	ref	41 (7.98)	ref
T2	-10.84 (-56.39, 34.70)	-0.02 (-0.18, 0.14)	39 (7.63)	1.01 (0.63, 1.61)
T3	-63.02 (-108.72, -17.32)	-0.13 (-0.28, 0.03)	50 (9.75)	1.33 (0.85, 2.07)
*P* for trend	0.004	0.101		0.160

*Abbreviations*: *DON* deoxynivalenol, *fDON* total DON, *tDON* free DON, *OR* odds ratio, *CI* confidence interval

Adjusted Model: adjusted for age, pre-pregnancy BMI, weight, average personal income, education attainment, parity, abnormal pregnancy-labor history, morning sickness, alcohol intake, smoking, season of sample collection, infant sex, and gestational age at delivery

### The associations between PDI and birth outcomes

Table 3 presents the associations between maternal provisional daily intake of DON and birth outcomes. For each unit increase in Ln-PDI, birth weight decreased by 23.69 g (95% CI: -38.99, -8.39). Compared to the lowest PDI tertile, the highest PDI tertile was associated with a significant decrease in birth weight (adjusted β = -82.88 g, 95% CI: -129.10, -36.66) and birth length (adjusted β = -0.17 cm, 95% CI: -0.33, -0.01). When compared with those whose PDI does not exceed PMTDI (PDI < 1), our results indicated that exceeding PMTDI (PDI ≥ 1) was inversely associated with birth weight (adjusted β: -79.79 g; 95% CI: -119.09, -40.49), and birth length (adjusted β: -0.21 cm; 95% CI: -0.34, -0.07).

**Table 3:** Associations of maternal provisional daily intake (PDI) of DON with birth outcomes

	**Birth weight (g)**	**Birth length (cm)**	**Small for gestational age**	
	β (95%CI)	β (95%CI)	Case (%)	OR (95%CI)
**PDI**				
Ln-PDI	-23.69 (-38.99, -8.39)	-0.03 (-0.08, 0.02)	130 (8.45)	1.10 (0.94, 1.28)
Tertiles				
T1	ref	ref	35 (6.84)	ref
T2	-23.99 (-69.98, 22.00)	0.02 (-0.14, 0.18)	41 (7.99)	1.23 (0.77, 1.98)
T3	-82.88 (-129.10, -36.66)	-0.17 (-0.33, -0.01)	54 (10.53)	1.62 (1.03, 2.55)
* P* for trend	< 0.001	0.016		0.036
Dichotomous value				
< PMTDI	ref	ref	72 (7.30)	ref
≥ PMTDI	-79.79 (-119.09, -40.49)	-0.21 (-0.34, -0.07)	58 (10.51)	1.48 (1.02, 2.15)

*Abbreviations*: *PDI* provisional daily intake, *DON* deoxynivalenol, *PMTDI* provisional maximum tolerable daily intake, *OR* odds ratio, *CI*, confidence interval

Adjusted Model: adjusted for age, pre-pregnancy BMI, average personal income, education attainment, parity, abnormal pregnancy-labor history, morning sickness, alcohol intake, smoking, season of sample collection, infant sex, and gestational age at delivery

The adjusted OR for per unit increase in Ln-PDI was 1.10 (95% CI: 0.94, 1.28) for SGA, but there was not statistically significant. However, pregnant women in the highest tertile of PDI displayed about 1.62-fold increased risk of SGA (adjusted OR = 1.62; 95% CI: 1.03, 2.55; *P* for trend = 0.036) compared with women who were in the lowest tertile. In addition, women whose PDI exceeds PMTDI were more likely to deliver an SGA baby (OR = 1.48; 95% CI: 1.02, 2.15). We did not find any relationships between provisional daily DON intake and LBW or PTB (Additional file 1: Table S4).

### Stratified and sensitivity analyses

**Fig. 1:**
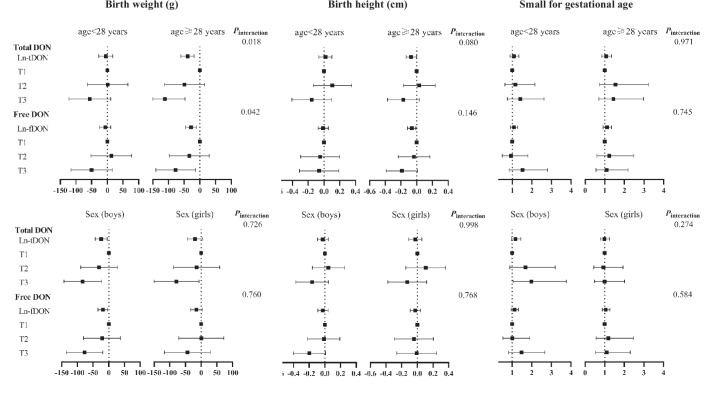
Stratified analyses of the associations of maternal urinary DON levels during pregnancy with birth outcomes. The β (95% CI) and OR (95% CI) are from linear regression models and logistic regression models, adjusted for age, pre-pregnancy BMI, weight, average personal income, education attainment, parity, abnormal pregnancy-labor history, morning sickness, alcohol intake, smoking, season of sample collection, infant sex, and gestational age at delivery

**Fig. 2:**
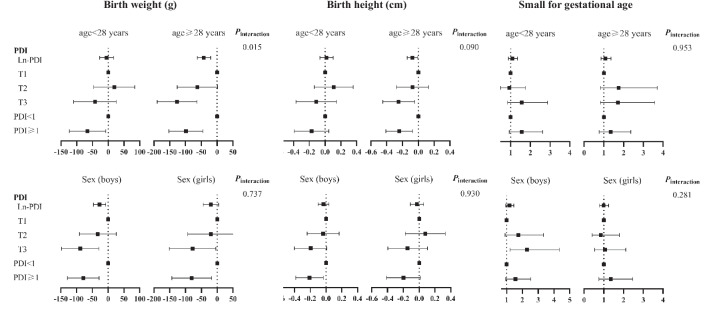
Stratified analyses of the associations of maternal provisional daily intake of DON with birth outcomes. The β (95% CI) and OR (95% CI) are from linear regression models and logistic regression models, adjusted for age, pre-pregnancy BMI, average personal income, education attainment, parity, abnormal pregnancy-labor history, morning sickness, alcohol intake, smoking, season of sample collection, infant sex, and gestational age at delivery

The stratified analysis revealed somewhat stronger associations among elderly pregnant women (age ≥ 28 years) or baby boys, the interaction between maternal age and maternal DON exposure (tDON, fDON, and PDI) on birth weight was statistically significant (Fig. 1 and Fig. 2). Similar results were found in the analysis of the association between DON levels and birth outcomes after further adjustment for cereal intake in women with available dietary data (Table 4 and 5).

**Table 4:** The associations between urinary DON levels (ng/mL) and birth outcomes among pregnant women with available dietary data (*n* = 712)

	**Birth weight (g)**	**Birth length (cm)**	**Small for gestational age**	
	β (95%CI)	β (95%CI)	Case (%)	OR (95%CI)
**Total DON**				
Ln-tDON	-28.78 (-50.59, -6.97)	-0.05 (-0.13, 0.03)	66 (9.27)	1.12 (0.90, 1.39)
T1	ref	ref	21 (8.50)	ref
T2	6.10 (-57.26, 69.43)	0.16 (-0.08, 0.40)	18 (7.17)	0.89 (0.45, 1.75)
T3	-96.50 (-163.04, -29.96)	-0.26 (-0.51, -0.01)	27 (12.62)	1.67 (0.89, 3.14)
* P* for trend	0.002	0.011		0.061
**Free DON**				
Ln-fDON	-15.83 (-33.14, 1.48)	-0.05 (-0.12, 0.01)	66 (9.27)	1.04 (0.88, 1.23)
T1	ref	ref	23 (9.58)	ref
T2	-5.84 (-58.33,70.01)	-0.05 (-0.30, 0.19)	18 (7.09)	0.73 (0.38, 1.43)
T3	-68.87 (-135.71, -2.03)	-0.21 (-0.46, 0.05)	25 (11.47)	1.27 (0.68, 2.37)
*P* for trend	0.021	0.096		0.254

*Abbreviations*: *DON* deoxynivalenol, *fDON* free DON, *tDON* total DON, *OR* odds ratio, *CI* confidence interval

Adjusted Model: adjusted for age, pre-pregnancy BMI, weight, average personal income, education attainment, parity, abnormal pregnancy-labor history, morning sickness, alcohol intake, smoking, season of sample collection, infant sex, gestational age at delivery, and cereal intake

**Table 5:** The associations between PDI and birth outcomes among pregnant women with available dietary data (*n* = 712)

	**Birth weight (g)**	**Birth length (cm)**	**Small for gestational age**	
	β (95%CI)	β (95%CI)	Case (%)	OR (95%CI)
**PDI**				
Ln-PDI	-30.79 (-52.94, -8.64)	-0.06 (-0.14, 0.03)	66 (9.27)	1.13 (0.91, 1.41)
Tertiles				
T1	ref	ref	19 (7.88)	ref
T2	-10.62 (-74.94, 53.70)	0.05 (-0.19, 0.30)	18 (7.11)	0.94 (0.47, 1.86)
T3	-112.24 (-179.54, -44.94)	-0.31 (-0.57, -0.06)	29 (13.30)	1.78 (0.95, 3.34)
* P* for trend	< 0.001	0.005		0.036
Dichotomous value				
< PMTDI	ref	ref	36 (7.56)	ref
≥ PMTDI	-103.69 (-160.99, -46.39)	-0.36 (-0.57, -0.14)	30 (12.71)	1.71 (1.01, 2.91)

Abbreviations: *PDI *provisional daily intake, *DON* deoxynivalenol, *PMTDI* provisional maximum tolerable daily intake, *OR* odds ratio, *CI* confidence interval

Adjusted Model: adjusted for age, pre-pregnancy BMI, average personal income, education attainment, parity, abnormal pregnancy-labor history, morning sickness, alcohol intake, smoking, season of sample collection, infant sex, gestational age at delivery, and cereal intake

## Discussion

The present study examined the urinary DON levels during the second trimester and estimated the PDI of DON in Chinese pregnant women. We found that about 35.9% of them exceeded the PMTDI value set by JECFA (1 μg/kg bw). Notably, this is the first epidemiological study to suggest a possible dose–response relationship between DON exposure and adverse birth outcomes. Specifically, our findings demonstrated that maternal urinary DON levels and provisional daily DON intake were positively associated with lower birth weight. Furthermore, pregnant women with a PDI of DON exceeding the PMTDI had a 1.48-fold increased risk of delivering SGA offspring.

The levels of DON among pregnant women in the present study were moderately high compared to those in other countries. More than 90% of pregnant women in our study had detectable levels of total DON in their urine, with a median concentration of 12.1 ng/mL or 13.5 ng/mg creatinine, indicating relatively high DON levels. In a study conducted in Bangladesh, only about half of the pregnant women had detectable levels of DON, with mean concentrations of 0.86 ng/mL or 2.14 ng/mg creatinine [26]. Another study among pregnant women in Egypt reported a DON detection rate of 68% with a geometric mean of 2.8 μg/g creatinine [27]. In Bradford, UK, Hepworth et al. analyzed urine samples of pregnant women in the third trimester for DON analysis and found that urinary DON was detected in all measured samples, with a geometric mean of 10.3 ng/mg creatinine [28], which is similar to the detection level in our research. However, pregnant women from Croatia had significantly higher DON levels than our study population, with a median concentration of 48.7 ng/mL or 41.2 ng/mg creatinine, and an estimated DON intake of 2.5 μg/kg bw/day, with 48% of the subjects exceeding the PMTDI [29]. The mean and median estimated dietary DON exposure for our study population was 1.0 μg/kg bw and 0.7 μg/kg bw, respectively. Moreover, 35.9% of pregnant women exceeded the PMTDI, indicating a potential hazard for high DON exposure in pregnant women from Wuhan, Hubei province, China. Climatic conditions are more conducive to fungi growth and toxin reproduction in China, which is located in a temperate zone [30]. Moreover, the dietary pattern of the Chinese is mainly grain-based food consumption, which may cause higher dietary DON exposure [11].

Currently, only one cohort study has evaluated the association between serum DON levels and adverse birth outcomes in 483 pregnant women at 8–24 weeks of gestation in rural Ethiopia. However, this study did not find a statistically significant association [31]. The serum DON levels were relatively low, detected in only 38.7% of the population, with concentrations ranging from 0–2.4 ng/mL. In Nanjing, China, Fan et al. detected multiple mycotoxins in paired plasma and urine samples and found that the detection rate and mean concentration of DON in urine were higher than those in plasma [32]. Therefore, a larger sample size and higher detection levels in our study may contribute to finding statistically significant associations between urinary DON levels and adverse birth outcomes.

Our findings reveal that dietary DON exposure during pregnancy may lead to the exposure of DON in early life, resulting in fetal growth retardation. Smaller size or relative thinness at birth and during infancy have been associated with increased rates of coronary heart disease, stroke, type 2 diabetes mellitus, adiposity, metabolic syndrome, and osteoporosis in adult life [33]. So given this reality, it is important to be concerned about the relationship between maternal DON exposure and their offspring. In addition, more research is needed to determine the safe threshold for DON exposure in pregnant women, and stricter policies should be developed to strengthen food safety.

There are several possible biological mechanisms that may explain the effect of DON on growth faltering. The first is that DON may interfere with the nutritional efficiency of maternal intestinal absorption, which could affect fetal weight gain [34, 35]. Even at low doses, chronic consumption of DON-contaminated food can induce intestinal inflammation and damage the intestinal mucosa [36, 37]. The toxicity of DON is mainly through the activation of mitogen-activated protein kinases signaling pathway and the expression of genes that alter essential physiological and immune functions of tissues, which reduces the expression of tight junction proteins such as claudins and decreases intestinal barrier permeability [38]. In addition, studies have shown that dietary DON exposure may affect intestinal health by destroying the balance and diversity of commensal microbiota, leading to ecological imbalance [39, 40]. Another potential mechanism may be related to changes in growth hormone levels controlled by insulin-like growth factors [41, 42], which are mainly produced in the liver. Thus, DON-induced hepatotoxicity may be a crucial determinant of growth and development [43].

Several limitations in the present study should be mentioned. First, though we performed a multivariate analysis model to adjust for potential confounders, there were unmeasured confounders that could affect exposure concentrations and infant birth outcomes. Second, we only measured DON in the urine but not in the blood. Studies have demonstrated that urinary DON biomarkers have been shown to be a good indicator of the body’s burden to assess DON exposure [44, 45]. Third, pregnant women provided urinary in the second trimester were included for analyses in the present study. Dietary changes due to early trimester pregnancy symptoms may result in urinary DON levels that do not accurately reflect DON exposure during pregnancy. Finally, we focused on a single mycotoxin exposure without considering the potential effects of other mycotoxins exposure. Pregnant women may be exposed to multiple mycotoxins simultaneously by dietary, since similar environmental conditions could produce several mycotoxins in foods at the same time [46]. Although a cohort study in Bangladesh reported that maternal dietary intake of ochratoxin A was associated with higher risk of having an LBW baby, it did not find a difference between exposure alone and combined exposure to other mycotoxins [47]. Therefore, further epidemiological studies are needed to evaluate the complex effects of co-exposure of mycotoxins on the health risk in pregnant women.

## Conclusions

In summary, higher DON exposure during pregnancy may be associated with lower birth weight and birth length, and a higher risk of SGA in their offspring. More attention and measures for policy maker are needed to reduce deoxynivalenol exposure in Chinese pregnant women.

### Supplementary Information

**Additional file 1**:** Fig.S1. **Flowchart of the study population for analysis. **Fig.S2.** Scatterplot of urinary free DON (fDON) and urinary total DON (tDON), all in logarithmic scale (*r* = 0.810, *P *< 0.001). **Table S1.** Maternal and neonatal characteristics for the study population, urine available and unavailable subjects.^a^.** Table S2.** Associations of maternal urinary creatinine-corrected DON levels (ng/mg Creatinine) during pregnancy with birth outcomes. **Table S3.** Associations of maternal urinary DON levels (ng/mL) during pregnancy with the risk of low birth weight and preterm birth. **Table S4.** Associations of maternal urinary DON levels (ng/mL) during pregnancy with the risk of low birth weight and preterm birth.

## Data Availability

Data are not publicly available due to ethical restrictions but can be obtained from the corresponding authors upon reasonable request and subject to appropriate approvals, including from the TMCHC cohort’s Executive Committee.

## Abbreviations

DON: Deoxynivalenol
PMTDI: Provisional maximum tolerable daily intake
Body weight: Bw
JECFA: Joint Expert Committee on Food and Additives
EFSA: European Food Safety Authority
TMCHC: Tongji Maternal and Child Health Cohort
fDON: Free DON
tDON: Total DON
UPLC-MS/MS: Ultra-performance liquid chromatography-tandem mass spectrometry
LOD: Limit of detection
PDI: Provisional daily intake
SGA: Small for gestational age
LBW: Low birth weight
PTB: Preterm birth
BMI: Body mass index
SD: Standard deviation
CI: Confidence interval
OR: Odds ratio
