# Supplementary material for: Adverse associations between maternal deoxynivalenol exposure and birth outcomes: a prospective cohort study in China
Source: BMC Med. 2023 Aug 28;21:328. doi: 10.1186/s12916-023-03011-5 (PMC10464359; doi:10.1186/s12916-023-03011-5)
Supplement: Supplementary file 1 — Additional file 1: Fig.S1. Flowchart of the study population for analysis. Fig.S2. Scatterplot of urinary free DON (fDON) and urinary total DON (tDON), all in logarithmic scale (r = 0.810, P < 0.001). Table S1. Maternal and neonatal characteristics for the study population, urine available and unavailable subjects.a. Table S2. Associations of maternal urinary creatinine-corrected DON levels (ng/mg Creatinine) during pregnancy with birth outcomes. Table S3. Associations of maternal urinary DON levels (ng/mL) during pregnancy with the risk of low birth weight and preterm birth. Table S4. Associations of maternal urinary DON levels (ng/mL) during pregnancy with the risk of low birth weight and preterm birth. [file 12916_2023_3011_MOESM1_ESM.docx]

7570 pregnant women with singleton live births followed up to delivery were included

2844 pregnant women providing urine sample ≤ 28 weeks of gestation

Failed to provide urine samples before 28 weeks of gestation (N=4726)

Provided urine sample <16 weeks of gestation (N=1306)

1538 women eligible

8649 pregnant women between 0~16 gestational weeks were enrolled TMCHC

Lost to follow up during pregnancy (N=691)

Stillbirths and miscarriage (N=222)

Twins and multiple pregnancy (N=166)

**Fig.S1** Flowchart of the study population for analysis.


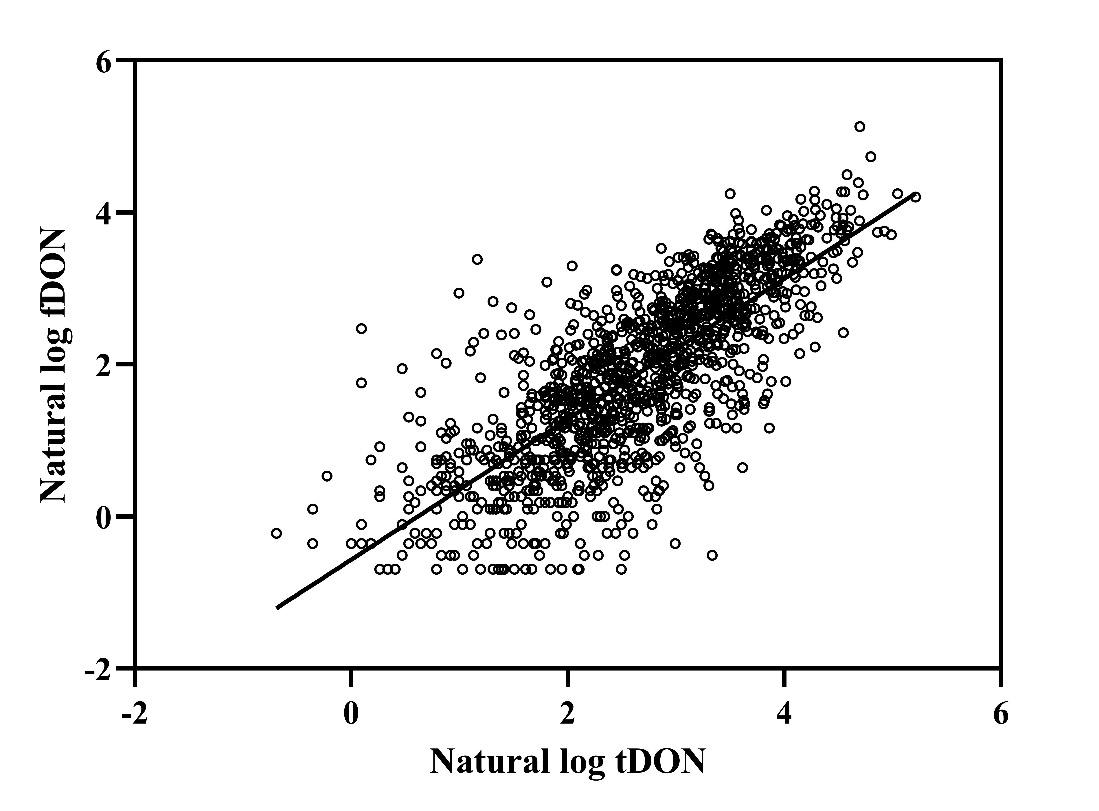


**Fig.S2** Scatterplot of urinary free DON (fDON) and urinary total DON (tDON), all in logarithmic scale (*r* = 0.810, *P* < 0.001).

**Table S1** Maternal and neonatal characteristics for the study population, urine available and unavailable subjects. ^a^

|  | Study population ^b^ | Urine available subjects ^c^ | Urine unavailable subjects ^d^ |
| --- | --- | --- | --- |
| N | 1538 | 2844 | 4726 |
| **Maternal characteristics** |  |  |  |
| Maternal age (years) | 28.4 ± 3.7 | 28.3 ± 3.6 | 28.1 ± 3.5 |
| BMI (kg/m2) | 20.9 ± 2.9 | 20.9 ± 2.8 | 20.7 ± 2.8 |
| Weight (kg) | 54.8 ± 7.9 | 54.7 ± 8.0 | 55.1 ± 8.4 |
| Average personal income (RMB yuan/month) |  |  |  |
| <5000 | 458(33.2) | 991 (34.0) | 2106 (44.5) |
| 5000-9999 | 600(43.5) | 1275 (43.7) | 1710 (36.1) |
| ≥10000 | 305(22.1) | 609 (20.9) | 672 (14.2) |
| Missing | 15(1.1) | 41 (1.4) | 246 (5.2) |
| Education attainment (years) |  |  |  |
| ≤12 | 235(17.1) | 448 (15.4) | 791 (16.7) |
| 13-15 | 381(27.7) | 769 (26.4) | 1311 (27.7) |
| ≥16 | 732(53.1) | 1618 (55.5) | 2472 (52.2) |
| Missing | 30(2.2) | 81 (2.8) | 159 (3.4) |
| Parity (Primiparous) | 1112(80.7) | 2383 (81.7) | 4042 (85.4) |
| Abnormal Pregnancy-Labor History (Yes) | 513(37.2) | 1083 (62.9) | 1705 (36.0) |
| Morning sickness (Yes) | 1152(83.6) | 2428 (83.3) | 3716 (78.5) |
| Smoking (Yes) | 103(7.5) | 202 (6.9) | 467 (10.2) |
| Alcohol intake (Yes) | 51 (3.7) | 122 (4.2) | 205 (4.5) |
| **Infant characteristics** |  |  |  |
| Sex (male) | 694 (53.1) | 1580 (55.6) | 2490 (52.6) |
| Gestational age (weeks) | 39.1 ± 2.4 | 39.3 ± 2.0 | 39.3 ± 1.4 |
| Birth weight (g) | 3331.1 ± 444.8 | 3331.1 ± 445.1 | 3328.5 ± 445.9 |
| Birth Length (cm) | 50.1 ± 1.5 | 50.1 ± 1.5 | 50.1 ± 1.5 |

^a^ Data are presented as mean ± SD, median (95% range), or number (%).

^b^ Participants retained in the present study.

^c^ Pregnant women with singleton live births who provided urine samples before 28 weeks.

^d^ Pregnant women with singleton live births who failed to provide urine samples before 28 weeks.

**Table S2** Associations of maternal urinary creatinine-corrected DON levels (ng/mg Creatinine) during pregnancy with birth outcomes.

|  | **Birth weight (g)** |  | **Birth length (cm)** |  | **Small for gestational age** | |
| --- | --- | --- | --- | --- | --- | --- |
|  | β(95%CI) |  | β(95%CI) |  | Case(%) | OR(95%CI) |
| **Total DON** |  |  |  |  |  |  |
| Ln-tDON | -14.80 (-32.80, 3.21) |  | -0.01 (-0.07, 0.05) |  | 130 (8.45) | 1.06 (0.89, 1.26) |
| T1 | ref. |  | ref. |  | 36 (7.03) | ref. |
| T2 | -46.64 (-92.34, -0.94) |  | -0.03 (-0.19, 0.13) |  | 45 (8.77) | 1.34 (0.84, 2.13) |
| T3 | -50.00 (-95.82, -4.17) |  | -0.11 (-0.27, 0.05) |  | 49 (9.61) | 1.42 (0.90, 2.25) |
| *P* for trend | 0.062 |  | 0.165 |  |  | 0.183 |
| **Free DON** |  |  |  |  |  |  |
| Ln-fDON | -14.11 (-28.11, -0.10) |  | -0.03 (-0.08, 0.02) |  | 130 (8.45) | 1.09 (0.95, 1.25) |
| T1 | ref. |  | ref. |  | 41 (8.01) | ref. |
| T2 | -27.74 (-73.31, 17.82) |  | -0.10 (-0.26, 0.06) |  | 45 (8.77) | 1.16 (0.74, 1.82) |
| T3 | -26.54 (-72.28, 19.20) |  | -0.04 (-0.20, 0.12) |  | 44 (8.58) | 1.07 (0.68, 1.69) |
| *P* for trend | 0.35 |  | 0.809 |  |  | 0.892 |

Abbreviations: DON, deoxynivalenol; fDON, total DON; tDON, free DON; OR, odds ratio; CI, confidence interval.

Adjusted Model: adjusted for age, pre-pregnancy BMI, weight, average personal income, education attainment, parity, abnormal pregnancy-labor history, morning sickness, alcohol intake, smoking, season of sample collection, infant sex, and gestational age at delivery.

**Table S3** Associations of maternal urinary DON levels (ng/mL) during pregnancy with the risk of low birth weight and preterm birth.

|  | **Low birth weight ^a^** | |  | **Preterm birth ^b^** | |
| --- | --- | --- | --- | --- | --- |
|  | Case(%) | OR(95%CI) |  | Case(%) | OR(95%CI) |
| **Total DON** |  |  |  |  |  |
| Ln(tDON) | 37 (2.41) | 0.83 (0.61, 1.13) |  | 61 (3.97) | 0.96 (0.78, 1.19) |
| T1 | 17 (3.32) | ref. |  | 20 (3.91) | ref. |
| T2 | 8 (1.55) | 0.29 (0.08, 1.03) |  | 18 (3.49) | 0.65 (0.32, 1.29) |
| T3 | 12 (2.35) | 0.63 (0.23, 1.69) |  | 23 (4.51) | 1.14 (0.62, 2.10) |
| P for trend |  | 0.620 |  |  | 0.624 |
| **Free DON** |  |  |  |  |  |
| Ln(fDON) | 37 (2.41) | 1.03 (0.78, 1.37) |  | 61 (3.97) | 1.11 (0.93, 1.33) |
| T1 | 11 (2.14) | ref. |  | 16 (3.11) | ref. |
| T2 | 11 (2.15) | 0.57 (0.17, 1.84) |  | 21 (4.11) | 1.28 (0.65, 2.51) |
| T3 | 15 (2.92) | 1.26 (0.46, 3.48) |  | 24 (4.68) | 1.43 (0.74, 2.77) |
| P for trend |  | 0.404 |  |  | 0.341 |

Abbreviations: DON, deoxynivalenol; fDON, total DON; tDON, free DON; OR, odds ratio; CI, confidence interval.

^a^ Adjusted Model: adjusted for age, pre-pregnancy BMI, weight, average personal income, education attainment, parity, abnormal pregnancy-labor history, morning sickness, alcohol intake, smoking, season of sample collection, infant sex, and gestational age at delivery.

^b^ Adjusted Model: adjusted for age, pre-pregnancy BMI, weight, average personal income, education attainment, parity, abnormal pregnancy-labor history, morning sickness, alcohol intake, smoking, season of sample collection, and infant sex.

**Table S4** Associations of maternal urinary DON levels (ng/mL) during pregnancy with the risk of low birth weight and preterm birth.

|  | **Low birth weight ^a^** | |  | **Preterm birth ^b^** | |
| --- | --- | --- | --- | --- | --- |
|  | Case(%) | OR(95%CI) |  | Case(%) | OR(95%CI) |
| **PDI** |  |  |  |  |  |
| Ln(PDI) | 37 (2.41) | 0.84 (0.62, 1.15) |  | 61 (3.97) | 0.96 (0.78, 1.18) |
| Tertiles |  |  |  |  |  |
| T1 | 16 (3.13) | ref. |  | 19 (3.71) | ref. |
| T2 | 10 (1.95) | 0.34 (0.10, 1.10) |  | 21 (4.09) | 1.15 (0.60, 2.19) |
| T3 | 11 (2.14) | 0.60 (0.22, 1.63) |  | 21 (4.09) | 1.17 (0.61, 2.34) |
| p for trend |  | 0.519 |  |  | 0.692 |
| Dichotomous value |  |  |  |  |  |
| <PMTDI | 25 (2.54) | ref. |  | 39 (3.96) | ref. |
| ≥PMTDI | 12 (2.17) | 1.02 (0.41, 2.50) |  | 22 (3.99) | 1.02 (0.59, 1.76) |

Abbreviations: PDI, provisional daily intake; DON, deoxynivalenol; PMTDI, provisional maximum tolerable daily intake; OR, odds ratio; CI, confidence interval.

^a^ Adjusted Model: adjusted for age, pre-pregnancy BMI, average personal income, education attainment, parity, abnormal pregnancy-labor history, morning sickness, alcohol intake, smoking, season of sample collection, infant sex, and gestational age at delivery.

^b^ Adjusted Model: adjusted for age, pre-pregnancy BMI, average personal income, education attainment, parity, abnormal pregnancy-labor history, morning sickness, alcohol intake, smoking, season of sample collection, and infant sex.
